# Precision Medicine Approaches with Metabolomics and Artificial Intelligence

**DOI:** 10.3390/ijms231911269

**Published:** 2022-09-24

**Authors:** Elettra Barberis, Shahzaib Khoso, Antonio Sica, Marco Falasca, Alessandra Gennari, Francesco Dondero, Antreas Afantitis, Marcello Manfredi

**Affiliations:** 1 Department of Translational Medicine, University of Piemonte Orientale, 28100 Novara, Italy; 2 Center for Translational Research on Autoimmune and Allergic Diseases, University of Piemonte Orientale, 28100 Novara, Italy; 3 Department of Pharmaceutical Sciences, University of Piemonte Orientale, 28100 Novara, Italy; 4 Humanitas Clinical and Research Center, IRCCS, 20089 Rozzano, Italy; 5 Metabolic Signaling Group, Curtin Medical School, Curtin University, Perth 6845, Australia; 6 Department of Sciences and Technological Innovation, University of Piemonte Orientale, 15100 Alessandria, Italy; 7 NovaMechanics Ltd., Digeni Akrita 51, Nicosia 1070, Cyprus

**Keywords:** metabolomics, artificial intelligence, machine learning, precision medicine, biomarkers

## Abstract

Recent technological innovations in the field of mass spectrometry have supported the use of metabolomics analysis for precision medicine. This growth has been allowed also by the application of algorithms to data analysis, including multivariate and machine learning methods, which are fundamental to managing large number of variables and samples. In the present review, we reported and discussed the application of artificial intelligence (AI) strategies for metabolomics data analysis. Particularly, we focused on widely used non-linear machine learning classifiers, such as ANN, random forest, and support vector machine (SVM) algorithms. A discussion of recent studies and research focused on disease classification, biomarker identification and early diagnosis is presented. Challenges in the implementation of metabolomics–AI systems, limitations thereof and recent tools were also discussed.

## 1. Introduction

### 1.1. Machine Learning and Metabolomics

The use of metabolomics for personalized medicine is a rapidly emerging field. This growth has been supported by recent technological innovations that allow for the quantitative analysis of hundreds to thousands of molecules in a single sample and the application of advanced statistical and classification techniques. To deal with a large number of variables, feature selection and classification methods such as partial least-squares discriminant analysis (PLS-DA), principal component analysis (PCA), and machine learning have been largely employed [1,2,3,4,5]. The use of these methods is especially necessary for diagnostic models [6,7].

Machine learning, which is a sub-domain of artificial intelligence, can be divided into three categories: (1) supervised learning, (2) unsupervised learning, and (3) semi-supervised learning. Supervised machine learning algorithms utilize intensively statistical approaches to train a model on labeled data and make predictions about unknown (unlabeled) data. By contrast, unsupervised models deal with unlabeled data for training. Machine learning is the most ideal application for mass spectrometry data owing to its ability to cope with both linear and non-linear data. However, using machine learning for mass spectrometry applications is not a novel concept; an early study in the 1990s demonstrated the performance of artificial neural networks (ANNs) in classifying mass spectra [8]. Afterward, many other supervised algorithms were applied to mass spectrometry data to improve the classification of mass spectra [9]. In the twenty-first century, mass spectrometry diagnostic research using machine learning began to grow.

**Figure 1:**
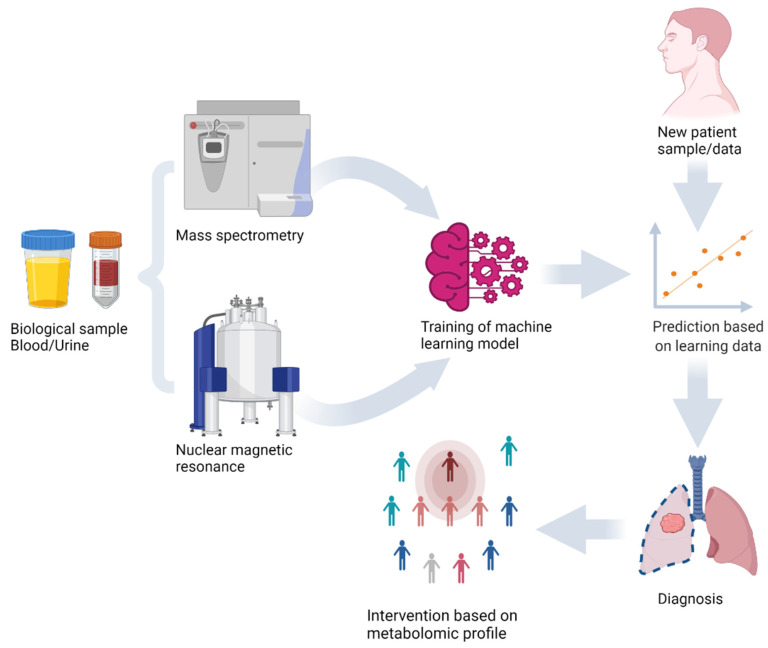
Machine learning model training and prediction of new sample using metabolomics analysis of biological samples with mass spectrometry and nuclear magnetic resonance.

In the early 21st century, one of the most significant studies on the combination of machine learning and mass spectrometry data showed early promise for disease diagnosis [10]. The ability of ANNs to measure protein expression levels in cancer patients was investigated. Authors obtained 98% accuracy using surface-enhanced laser desorption/ionization time of flight mass spectrometry technology [10]. Several other studies [11,12,13] have demonstrated that machine learning algorithms can enable personalized therapy through the analysis of omics data (Figure 1).

Today, machine learning excels at object classification, reasoning, and complicated decision making. DeepMind’s AlphaGo recently demonstrated stunning performance, proving that machine learning algorithms can manage complicated decisions when applied to real-world circumstances [14]. For example, a study by Li et al. [15] showed that machine learning can be used to detect focal nodular hyperplasia and atypical hepatocellular carcinoma from ultrasonic data. The authors found that machine learning algorithms achieved higher scores when the feature selection was assisted by radiologists, suggesting the use of the algorithm to support clinical decision making.

**Figure 2:**
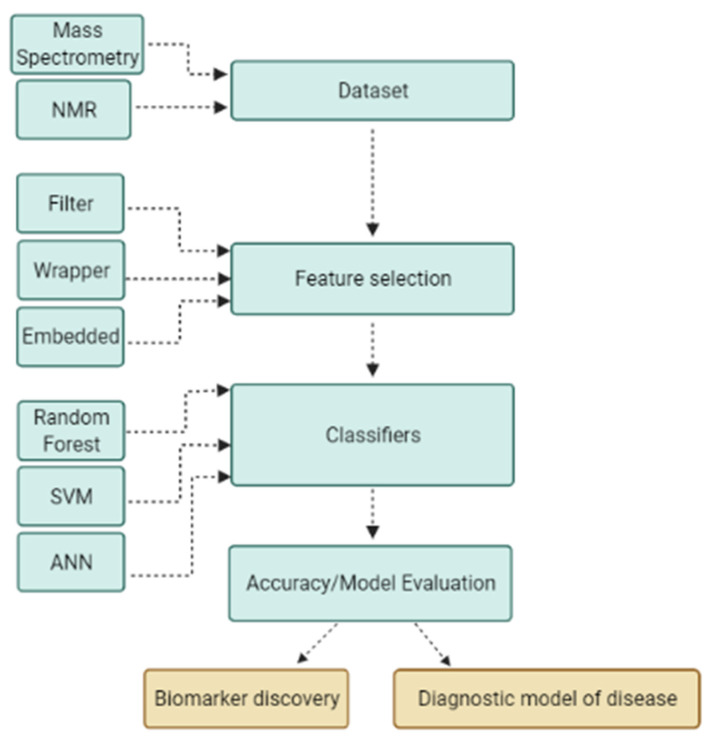
Machine learning application using a stepwise process for biomarker discovery and diagnostic modeling. The machine learning process begins with input of dataset generated by various platforms; data are then subjected to a feature selection algorithm to reduce dimensionality and obtain optimal subsets of features to build a robust classification model and to discover biomarkers.

Two well-known machine learning techniques for biomarker discovery and patient classification are feature selection algorithms and supervised classifier algorithms. The dataset must first be subjected to a feature selection approach in order to eliminate redundant and irrelevant information, as well as noise and computational burden. Following the feature selection step, which identifies the most discriminating subsets of features, a classifier algorithm is used to learn patterns from samples and predict unknown samples, which serves as the diagnostic model of disease. A machine learning application using a stepwise process for biomarker discovery and diagnostic modeling is illustrated in Figure 2.

In the following sections, we present and discuss the application of machine learning strategies to metabolomics data. In particular, we focus on widely used non-linear machine learning classifiers, such as ANN, random forest, and support vector machine (SVM) algorithms. Recently published reviews discussed the application of deep learning and non-linear machine learning algorithms mainly focusing on data processing of metabolomics data and on the identification of molecules including pathway analysis. Corsaro et al. focused their work more on a wide range of statistical approaches to elaborate NMR–metabolomics data from biomedical to food data, while Chen and coauthors discussed various strategies for expanding metabolite coverage, chemical derivatization, sample preparation, clinical disease markers, and machining learning for disease modeling [16,17]. However, the objective of the current review is to address the use of ML algorithms to obtain biomarkers and diagnostic models. A discussion on most recent tools for ML, on technical limitations and on future challenges is also reported. All the most recent strategies and approaches used to identify biomarkers and to classify patients based on metabolomic profiles of biofluids are reported and discussed.

### 1.2. Biomarkers

In biomedical science, there is a constant demand for new biomarkers to enable accurate diagnosis, therapy, and monitoring of the patient’s response. According to the National Institutes of Health Biomarkers Definitions Working Group, a biomarker is defined as “a characteristic that is objectively measured and evaluated as an indicator of normal biological processes, pathogenic processes, or pharmacologic responses to a therapeutic intervention” [18]. Body fluids such as urine, saliva, sputum, and sweat may include biomarkers that could be detected and investigated for indicators of normal or abnormal conditions. Emerging omics technologies for the quantification of thousands of molecules, together with the sophisticated development of artificial intelligence algorithms, can now accelerate biomarker discovery.

### 1.3. Metabolomics

Metabolomics is the large-scale study of small molecules such as bile acids, carbohydrates, amino acids, lipids, and organic acids, which are commonly known as metabolites. Metabolomics is particularly suitable for the characterization of biological samples since small molecules reflect the disease phenotype. In addition, the high reproducibility reached using internal standards, normalization procedures, and new instrumentations has allowed for highly accurate simultaneous analyses of hundreds of molecules in large-scale studies [19,20]. This has brought new challenges to precision medicine but also new opportunities, especially in the application of algorithms for data analysis. It has already been demonstrated that the use of multiple biomarkers can improve the accuracy of diagnostic tests [21]. The combination of metabolomics analysis and machine learning techniques could lead to the development of new approaches to perform more accurate and early diagnoses.

Metabolites can be used as biomarkers and to better understand the pathophysiology of many diseases [22]. Studies have found altered metabolites levels in diseased states [23,24]. The integration of metabolomic technology with machine learning can have the potential to greatly enhance diagnosis, identify more effective therapy targets, and permit more precise disease outcome prediction. Significant advances in technology, including techniques such as mass spectrometry, chromatography, and nuclear magnetic resonance (NMR) spectroscopy, have boosted the efficiency and reliability of metabolic profiling to the point that it is now recognized as a critical platform in the medical sector [25].

However, some limitations are present. Metabolomics data are characterized by intrinsic difficulties due to the nonlinear and linear correlations between metabolites, demanding constant algorithm refinement to obtain biological information. The tremendous complexity of metabolomics data (e.g., peak counts compared to samples), as well as missing values, batch effects during quantification, data noise production, and reproducibility, are all important issues. As a result, the metabolomics community is looking to machine learning approaches to overcome these obstacles [26,27].

## 2. Dimensionality and Features Selection

As previously discussed, metabolomics data include hundreds of features. Consequently, it is critical to reduce the dimensionality of the data to remove redundant and irrelevant information, improve the findings, and save computational time and resources. Three categories of feature selection algorithms are commonly found in the literature: filter, wrapper, and embedded methods [28].

### 2.1. Filter Methods

Filter methods are algorithm-independent and operate on statistical methods (e.g., ANOVA or Chi-squared tests). The dataset’s properties, such as the distance between two classes, and its statistical methodologies, such as correlations and coefficients, guide the filter method’s selection of features [29]. When compared to wrapper and embedded feature selection methods, filter techniques are faster and less computationally expensive. Nonetheless, filter feature selection methods have some disadvantages; for example, they do not take into account the interaction of features between them [30].

### 2.2. Wrapper and Embedded Methods

Unlike filter methods, wrapper methods use a predefined algorithm. The wrapper method searches through the feature space and generates an optimal feature subset through a search methodology, which is evaluated by training and testing criteria using a classifier algorithm. This procedure is performed until the selected feature subset satisfies the criteria. Nevertheless, although the wrapper technique has several benefits over the filter method, it also has some drawbacks. It is computationally expensive and difficult to implement with high-dimensional data. Additionally, selecting the best feature based on its predefined algorithm may increase the risk of overfitting [31]. Embedded feature selection differs from wrapper and filter methods in that the learner algorithm is not used by the filter method to select the best candidate, while it is used by the wrapper method to select the best features. However, unlike the filter and wrapper methods, the embedded system must carry out the process of learning and selecting features simultaneously, and thus selected features cannot be used by other classifiers [32].

The ideal feature selection method to build diagnostic models and to identify relevant biomarkers should be able to select the most discriminative features while minimizing the number of irrelevant ones. Today, thanks to the great availability of computational power, the use of hybrid or ensemble methods is strongly suggested. The potential of this last approach has been already demonstrated by several studies [33,34].

## 3. Machine Learning Applications for Biomarker Discovery

The process of identifying biomarkers using machine learning is not a simple task, as it entails various difficulties due to the correlation of variables. Numerous studies have shown the potential of feature selection algorithms to unravel the presence of disease biomarkers using metabolomics data. For example, researchers used random forest with a feature importance function to identify the most discriminating features to be used as potential biomarkers [35,36]. They calculated the importance score assigned to features according to the Gini index formula, and then they used the top-ranking features to train the classifier model.

Another study investigated the possibility of identifying biomarkers for lung cancer disease from plasma samples analyzed using targeted metabolomics. Fast correlation-based selection algorithms identified five top-performing biomarkers that could discriminate between healthy and lung cancer patients [37].

Bifarin et al. [38] performed urine sample analysis using liquid chromatography–mass spectrometry and NMR and designed a biomarker panel of 10 metabolites for predicting renal cell carcinoma status, utilizing recursive feature selection and the PLS regression algorithm. Features were chosen based on how often they appeared in both feature selection algorithms, and finally 10 metabolites were selected for training the classification model.

In another study, the authors evaluated the performance of multivariate methods with unbiased variable selection (MUVR) assessing serum samples analyzed by high-resolution mass spectrometry-based metabolomics. The MUVR approach selected 13 metabolites that produced excellent results using random forest, SVM, and logistic regression methods and established a panel of candidate biomarkers that have the ability to distinguish gout from asymptomatic hyperuricemia [39].

Furthermore, PLS-DA has been employed to determine the most discriminant lipids and metabolites to distinguish serum metabolomic and lipidomic profiles of patients with rheumatoid arthritis from healthy controls [35]. The authors proposed 26 molecules as candidate biomarkers for rheumatoid arthritis, and the chosen features were evaluated using three classifier algorithms: logistic regression, random forest, and SVM. In another study, 16 metabolites were obtained using recursive feature elimination combined with SVM (SVM-RFE) to discriminate epithelial ovarian cancer and age-matched control women. Samples were then projected to oPLS-DA containing 16 metabolites, for which classifier achieved 96.8% accuracy [40]. Table 1 presents recent diagnostic studies performed using metabolomics and machine learning.

In our experience, the selection of candidate biomarkers is the most important task, not only in developing a model for the diagnosis of a diseases, but also in describing the biological features that characterize that specific disorder. Although most of the reported research employed single strategies to select the most important variables, we think that the use of different algorithms to search for important markers might improve the machine learning performances. Another important factor that could affect the results is the number of selected biomarkers. In fact, the number of biomarkers could range from few to several tens. The definition of the optimal number of biomarkers is impossible to give, although we believe that a higher number of variables is always preferable. Indeed, metabolomics is particularly suitable to perform multi-analyte quantifications.

## 4. Application of Machine Learning for the Diagnosis of Diseases

Since the turn of the century, there has been a marked increase in the number of studies on metabolomics that have made use of machine learning techniques. Many studies have shown that machine learning can discriminate between healthy and disease, groups as well as identify important biomarkers for use in clinical decision making in a variety of settings [42,43]. The following sections present the most recent applications of supervised machine learning for the diagnosis of diseases.

### 4.1. Random Forest

Random forest is one of the most widely used supervised machine learning algorithms for mass spectrometry data due to its ability to cope with missing values, data noise, and reduced overfitting risk [44]. Random forest is a classification and regression technique that includes ensemble method of decision trees to predict classes. The algorithm establishes the outcome based on the predictions of the decision trees. For classification tasks, the output of the random forest is the class selected by most trees, where each tree can be described as an uncorrelated model.

Decision trees contain nodes which are structured in a hierarchical fashion from the top to the bottom, connected with branches. A logical query is present in each node, and it is sent downward via one of the two branches (binary split): the node is then linked to another node, proceeding in this manner until reaching the final node, which will ultimately determine the classification. Usually two-third of original samples are used for training of trees, while the remaining samples are utilized to evaluate the tree performance, in what are called OOBs (out-of-bag samples). While building the classification model, the rate at which OOB samples are misclassified across several classes, or the OOB error, can be used to quantify the prediction performance of the model. In the regression task, mean-squared error (MSE) metric can be used to calculate the average distance between OOB predictions and the actual continuous response variable.

Random forest was shown to be more efficient at diagnosing colorectal cancer (with 100% accuracy) using metabolomics data as well as choosing putative biomarkers, stability, prediction ability, and overfitting in comparison with other classifier models [45].

Melo et al. [35] showed the resilience and superior performance of random forest in contrast to other classifier models to identify Zika virus by employing metabolomics data, demonstrating that random forest outperforms other classifier models. The model was developed using 42 features and evaluated to differentiate between groups. Lima et al. [46] reported 97% accuracy in diagnosing paracoccidioidomycosis, using a combination of random forest and metabolomics data.

Recent research demonstrated the identification of malignant mesothelioma using random forest and metabolomics data with 92% accuracy in the validation dataset [47]. Twenty dysregulated features that distinguished the malignant mesothelioma group from others were investigated. Among the 20 features, biliverdin and bilirubin were shown to have diagnostic potential. Additionally, random forest ranked biliverdin as the fourth-most significant variable in terms of overall significance, demonstrating how random forest can be used to select the best potential biomarkers. However, study limitations, like a smaller number of classes and sample numbers, were also reported in order to get an accurate image of the diagnostic model.

In a study focusing on diagnosing irritable bowel syndrome, Fukui et al. [48] used a combination of logistic regression and random forest to obtain a better score in terms of sensitivity and specificity than if either of the algorithms had been performed individually. Other researchers trained four classifier models—a generalized linear regression model, PLS-DA, PCA linear discriminant analysis, and random forest—on metabolomics data [49]. The models were trained using two approaches. In the first approach, the models were trained using all the metabolites. In the second approach, the models were trained with only pre-selected metabolites. Random forest with pre-selected variables was the most effective model, with an AUC score of 72%.

Thanks to the availability of several free tools (see chapter 6), today, RF is largely used for the classification of patients based on metabolomic profile.

### 4.2. Support Vector Machine

Today, SVM classification is the most frequently used machine learning technique in precision medicine. SVM is a model that uses “support vectors” to construct a decision boundary (hyper-plane) in a high-dimensional feature space. Support vectors are data points that are positioned close to the hyperplane, and hence aid to optimize the hyperplane itself [50].

The objective of hyperplane is to maximize the distance between two classes, while placing as few data points as possible on the incorrect side of the decision boundary [51,52].

For a given training samples, a hyperplane is generated to maximize the distance, which can be mathematically defined as:

$$W^{T}X+b=0$$

where ***W*** is the weight matrix, ***X*** represents the dataset and b is constant term.

SVM can also be used to classify non-linear data through process called *kernel trick.* There are several types of kernel trick used for different problems such as the polynomial kernel, Gaussian kernel, Gaussian radial basis function (RBF), Laplace RBF, sigmoid kernel, hyperbolic tangent kernel, linear splines kernel in one dimension. Nonetheless, radial basis function (RBF) is the first choice among other kernels and it is also widely used for non-linear task in metabolomics.

Regarding the application of SVM, in a recent study, the algorithm was used as a classifier alongside random forest and logistic regression to identify gout from asymptomatic hyperuricemia. The author stated that SVM outperformed the other classifiers in terms of getting a higher area under curve score in the validation set, while random forest achieved higher score in the training set but lower in the test set, indicating overfitting by the classifier model [39].

Song et al. [53] used SVM to identify an early biomarker of diabetic cognitive impairments in a mouse model. SVM achieved 91.66% accuracy in identifying two groups of samples based on seven features, and the authors proposed biomarkers that play a role in pathogenesis, such as the metabolism of nicotinamide and glutathione as well as tryptophan and sphingolipids. In another recent study, SVM was used to classify benzylpenicillin and multidrug resistance in *Staphylococcus aureus* [54]. The authors performed matrix-assisted laser desorption/ionization–time of flight mass spectrometry to identify signature profiles of antibiotic resistance in *S. aureus* isolates. In terms of accuracy, specificity, and sensitivity, SVM showed better performance than random forest, multilayer neuron perceptron neural network, and naive Bayes classifiers.

Another interesting study used SVM with a radial basis function kernel and three feature selection algorithms for the diagnosis of intrauterine growth restriction [55]. The authors performed mass spectrometry and NMR analysis on cord blood serum, obtaining an AUC of 91% with correlation-based feature selection (CFS). In terms of sensitivity and specificity, the CFS-selected features achieved lower scores than those of other feature selection algorithms. The final model was built using overlapping features—including creatinine, acetyl carnitine, butyryl carnitine, three lysophosphatidylcholines, and a phosphatidylcholine—of the three feature selection algorithms, obtaining an accuracy of 88%.

Zheng et al. [56] used the least-squares SVM (LS-SVM) with three kernel functions—linear, polynomial, and radial basis—to diagnose major depression. In the test dataset, LS-SVM with the radial basis function outperformed the other kernel functions and achieved 96% accuracy. The classifier was built with glucose–lipid signaling features such as poly-unsaturated fatty acids, lipids with acetoacetate, very low-density lipids/low density lipids, lipids with N-acetyl, glucose, adipic acid, and sugars with amino acids.

As shown in all the reported research, SVM is a very attractive algorithm to perform precision medicine studies and to identify potential metabolic biomarkers. In addition, SVM is particularly suitable when the number of biological replicates or patients is low.

### 4.3. Artificial Neural Networks

Like the human brain, ANNs can handle complicated (non-linear) elements inside information and predict future situations. Humans learn by making modest alterations to synaptic interconnected neurons, whereas ANNs learn by adjusting the interconnections between the processing components that make up the network structure.

In 1943, McCulloch and Pitts [57] defined artificial neurons as a mathematical function developed by mimicking natural biological neuron processes. The number of hidden layers and neurons in each layer is proportional to the situation’s complexity. A vector of predictor variables, each represented by a node, is sent to the input layer by the external system. These data are then multiplied by a set of weights in the first hidden layer (hence modified). To produce an axon-like output, these products are added together and fed through a non-linear transfer function (sigmoid, hyperbolic tangent). Weights are modified in ANN-supervised learning so that they approximately represent each target as a nonlinear function of the inputs. The ability to iterate an approximation without knowing the precise shape of the ideal nonlinear function beforehand is a significant benefit of ANN learning. During the supervised process of learning, a series of case pairings are given to the network, each of which consists of a collection of input values and a target value. The goal is to optimize the weight values in such a way as to create an output for each situation that is as near to the actual class. Predictions are then compared with actual class and inaccuracy assessed to a cost function.

In a metabolomics study performed on plasma samples from Parkinson’s disease patients, ANNs with 13 input layers, seven hidden layer units, and an output layer with a back propagation algorithm were used for the diagnosis [58]. The neural network algorithm showed 97.14% accuracy in the detection of disease progression. However, one misdiagnosed case was also reported.

Another study profiled the metabolites in sputum from patients with lung cancer and age-matched smokers as a control using flow infusion electrospray ion mass spectrometry [59]. The metabolomic profiles were then analyzed using ANNs in order to perform the diagnosis of lung cancer, specifically small-cell lung cancer (SCLC) and non-small-cell lung cancer (NSCLC). The authors were able to classify SCLC with 100% specificity and 80% sensitivity. Six metabolites were identified as candidate biomarkers between the NSCLC and SCLC groups: phenylacetic acid, L-fucose, caprylic acid, acetic acid, propionic acid, and glycine.

ANNs were also used to elaborate metabolite abundances obtained from dried blood spots analyzed with direct infusion mass spectrometry [60]. The authors compared acute cerebral infarction and intracerebral hemorrhage. Using an external validation set, they achieved more than 70% accuracy in diagnosing intracerebral hemorrhage from acute cerebral infarction. They used ANNs with 11 units and 10 hidden layers of neurons, and they identified 11 significant metabolites using stepwise regression, on which the ANN model was trained.

Finally, ANNs were applied to predict a wide range of autoimmune diseases, including rheumatoid arthritis, thyroid disease, multiple sclerosis, vitiligo, psoriasis, and inflammatory bowel disease [61]. The authors performed targeted metabolomic analysis of serum total fatty acids using gas chromatography mass spectrometry. The metabolomic results were analyzed with an ANN model using two hidden layers of neurons and 11 variables. The ANN model obtained 76.2% accuracy in the classification of the groups. The features that made a major contribution to the development of the classifier model were cis-11-eicosenoic, lauric acid, erucic acid, cis-10-pentadecanoic acid, stearic acid, myristic acid, heptadecanoic acid, and palmitic acid. Table 2 presents recent studies performed using metabolomics and machine learning for the diagnosis of diseases.

Although today ANNs are widely used for classification purposes, non-linear models may not be appropriate for every type of research and the issue of interpretability of the models and of selected biomarkers remains a major challenge.

### 4.4. Other Supervised Learning Algorithms

Apart from random forest, SVM, and ANN, numerous other types of supervised algorithms have been applied to metabolomics data for the diagnosis of diseases. For example, Goutman et al. [62] compared regularized logistic regression (RLR) with elastic net regularization to random forest and SVM for the prediction of amyotrophic lateral sclerosis. The authors performed untargeted plasma metabolomics, and the best classification performances were obtained with RLR, with an AUC score of 98%. Another study used metabolomics data to identify lung cancer using five supervised machine learning algorithms: ada boost, ANN, naive Bayes, random forest, and SVM. In the training set, artificial neural networks and naive Bayes achieved 100% accuracy, however ANN failed to get a comparable result in the validation set. While using naive Bayes, authors were able to distinguish between healthy and lung tumor patients with 100% accuracy [37].

Huang et al. [63] proposed an interesting use of pathway-based metabolomic features as robust biomarkers for breast cancer. The authors developed a new computational method that uses personalized pathway dysregulation scores for disease diagnosis and applied the method, in combination with CFS and classification methods, to predict breast cancer occurrence. Three classifiers were compared, and the model was tested using data from plasma samples, serum samples, and age-matched breast cancer RNA-seq. In terms of accuracy, the logistic regression surpassed both random forest and SVM, and it achieved an AUC score of 98%. In the next step, the logistic regression was applied to the three remaining test datasets (external to the model), which included the 20% hold-out plasma testing samples, the complete serum sample collection, and the RNA-seq data from the TCGA, obtaining AUC scores of 0.9230, 0.9950, and 0.9946, respectively. Recently, a study was carried out with utilization of k-nearest neighbors (KNN), random forest and SVM with linear and nonlinear kernel support vector machine to predict the status of renal cell carcinoma. K-nearest neighbors outperformed other classifiers with Mass spectrometry generated metabolomics data with 10 selected metabolites and achieved accuracy of 81% with 96% AUC value [38].

Tong et al. [64] evaluated the performances of linear and non-linear models (e.g., logistic regression and SVM) using a variety of parameters for diagnosing interstitial cystitis (IC). In terms of diagnosing IC, the logistic regression model surpassed SVM with an AUC score of 90%. SVM, however, outperformed the logistic regression model with a polynomial kernel degree of 5. This research had several drawbacks, including an imbalanced class dataset (43 IC patients and 16 healthy controls) as well as a limited number of samples.

The k-nearest neighbors machine learning algorithm can be considered as a read-across strategy [65], as it requires experimental observations of only a few neighbors (similar patients metabolomics profile) of the query patients with unknown classification, in order to compute the endpoint of interest prediction. The KNN methodology is a “lazy” learning technique that classifies an instance based on the majority vote of the k-closest training examples (neighbors). EnaloskNN (developed by NovaMechanics as a new node in KNIME) [66] predicts the unknown endpoint based on the k (k = 1, 2, 3, …)-nearest neighbor’s characteristics in the training set, in the features space R_n_ where n is the total number of patients metabolomics profile used for the prediction. Where the endpoint has a numeric class, the prediction is the distance weighted average of the endpoint of the selected neighbors. An optimal k-value is selected based on the calculated Euclidean distance between all instances and as weighting factors the inverse distances are used [67,68,69]. In the case of a categorical endpoint each instance is assigned to the class indicated by the weighted majority vote of the k-closest neighbors. Another important aspect of the analysis—apart from the simple endpoint prediction—is the possibility to observe the groups of k-neighbors of each test patients, and therefore to specify and map the analogous space. This is a prerequisite of the read-across framework, and which can be used to support the justification of the read-across hypothesis. The EnaloskNN node has the benefit of providing not only the predictive results, but also the specific neighbors along with their Euclidian distances, as well allowing the visualization of the entire patient’s predictive space. Thus, this algorithm could be particularly suitable for the analysis of metabolomics data.

## 5. Factors Influencing Biomarker Selection through Machine Learning

Today, there are several concerns from metabolomic community about using machine learning algorithms, mainly due to the lack of explanation on the selection of biomarkers that a machine learning algorithm can provide. However, by using recently developed methods for interpretation of the ML model, for example LIME (local interpretable model-agnostic explanations) and Shapley additive explanation (SHAP) analysis, this issue can be partially addressed [70,71,72]. In a clinical setting, there are several aspects that must be considered to analyze the performances of a potential biomarker such as its positive or negative prediction value, the ROC (receiver operating characteristic curve) and AUC values [73]. Biomarker selection can be influenced by a variety of factors, like class imbalance, which can lead to uncorrected results. Fu. et al. proposed an approach based on the embedded feature selection method LASSO (least absolute shrinkage and selection operator), rather than using oversampling method to balance data [74]. The LASSO method was used also by Fukui et al. [48] on a class-imbalanced dataset composed of 85 patients with irritable bowels syndrome and 26 healthy subjects. Authors were able to obtain a high AUC score of 90% [48}. Another very important issue in metabolomics data-based biomarker discovery is the presence of missing values that can occur due to technical reason or biological origin [75,76]. The batch effect also influences the selection of biomarkers and can affect the prediction of the classifier model, as addressed by Kehoe et al. [77]. Indeed, the authors showed that when training and test data are collected within the same batch of analysis, the classification models are more accurate, but they failed to achieve the same level of accuracy with data generated in a different session of analysis. To address this problem, the authors developed a pipeline using an sSVM (sparse support vector machines) approach to sequester batch effects in the selection of biomarkers. Another restraint is the number of samples for training and validation of ML models. The metabolomics community has conducted a substantial number of studies using fewer than 100 samples [49,51,53]. Models built and validated on a small number of cases can raise the possibility of misdiagnosing individuals. This can be reduced by using a nested cross-validation approach [78].

## 6. Machine Learning Tools for Metabolomic Analysis

ML is advancing rapidly, and today offers a wide range of algorithms to handle challenging issues in the metabolomics field as well as to discover potential biomarkers. In the fields of NMR and mass spectrometry, ML has already been used to improve data-processing methods [79]. Many of the ML-based tools such as WEKA, KNIME, Orange data mining [80,81,82] have an intuitive user interface, no programming skills are required, and the tools are open source [83]. Other popular open-source libraries include Scikit-learn (also known as sklearn) for implementation of machine learning algorithms in Python [84], and TPOT, which provides an automated pipeline of machine learning algorithms that uses genetic programming stochastic global search approach to sort out top-performing ML models [85]. Caret library in R provides a plethora of machine learning algorithms and feature selection methods [86]. In addition, the use of ANN-based data analysis tools like PyTorch, Keras, and TensorFlow is growing in the metabolomics field [85]. However recently developed automated machine learning and deep learning pipelines such as AutoGluon, AutoPrognosis, H_2_O, PennAI have not yet been examined with metabolomics data [87,88]. In Table 3 are reported the most widely used tools and libraries in metabolomics studies.

## 7. Limitations

Despite advancements in machine learning in the health care sector, several obstacles remain unresolved. The first difficulty in developing a machine learning-aided diagnostic model using metabolomics is determining the minimum necessary data to represent a specific biological problem/disease. It is not easy to generate datasets that properly represent the population’s variance while at the same time including a sufficient number of samples for training and evaluating a robust model on an independent dataset. In addition, the quality of metabolomics data may vary according to experimental procedure biases. Often, machine learning models are trained on a small number of datasets, resulting in model failure when applied to more diverse data. Another problem related to machine learning models is the reproducibility of findings and the capacity of the algorithms to explain the results; as datasets get larger, it becomes more difficult to explain the logic behind algorithmic decisions [96]. Numerous algorithms are considered “black boxes” owing to the lack of information underpinning the mathematics of prediction. This aspect of machine learning is a major disadvantage when it comes to incorporating it into clinical decision making.

As shown in previous sections, several classifiers have been used for both biomarker discovery and the diagnosis of diseases. Random forest is usually less prone to overfitting, but it requires intensive computational power for huge datasets. On the other hands, SVM is very effective with high-dimensional data, but it can easily result in overfitting. Finally, ANNs are particularly suitable for huge datasets, but they are ineffective with small amounts of data.

## 8. Challenges in the Implementation of Metabolomics–AI Systems

The introduction of artificial intelligence in the clinic is a challenge that can be overcome only through the strong collaboration between clinicians, computational and laboratory scientists. In fact, artificial intelligence needs the access to large cohorts of patients, but also the biomarkers or the combination of markers should be measurable in a reproducible and easy manner. Lastly, the computational algorithms should be engineered in a user-friendly manner, especially in the setting of a busy practice.

The performances of a metabolomics–AI system strongly depend on the patients used to develop the models. The provenance of source data, such as patients from a different health system or a population different from the one used to develop AI models, can affect the diagnostic performances. To this aim, the use of large-scale multi-center cohorts can reduce the risk of using a poorly described panel of patients for the development of the AI model. In addition, all the clinical data should be considered while building the model. Another important aspect is to make the results clinically meaningful. The selection of appropriate patient-oriented endpoints and metrics to assess the performances of the model should be performed. This phase should be always developed focusing on the outcomes reported by clinicians. Knowing why an algorithm predicts a decision is very important for clinicians but also for patients, especially in case there is disagreement between human and AI prediction.

The challenge of delivering personalized care is also strictly correlated to the employment of adequate and reliable measurement of biomarkers. The analytical reproducibility is one of the most important issues for high-throughput technologies used for metabolomics analysis. This is particularly important when hundreds of samples are analyzed and elaborated using artificial intelligence algorithms. In fact, the quantification of molecules is affected by several factors such as the sample preparation, laboratory operators, the instrumentation used for the analysis, and biological variables. Although the use of spiked standards can reduce the sample preparation and instrumental variability, the reproducibility of untargeted metabolomic approaches is still limited, especially when different instruments are used. On the other hands, targeted metabolomics is characterized by a higher reproducibility, but at the same time the number of molecules that can be quantified is reduced, thus limiting its use in combination with AI algorithms.

Finally, the AI structure should be engineered in a manner that facilitates the integration of the AI models into the clinical decision, avoiding the use of stand-alone decision points. In addition, systems that require manual work to enter data should be avoided.

It is thus clear that the development of metabolomics–AI approaches for precision medicine and diagnostics is still far from the routine clinical use, although every year tens of research works on the development and application of pipelines that use statistical models for the diagnosis of diseases are published.

## 9. Conclusions

Artificial intelligence is a widely used approach in metabolomics and other high-throughput technologies, especially for performing early diagnosis. Recent studies have established several strategies for disease classification based on metabolomics profiles, demonstrating the potency of machine learning in the medical science field. Nonetheless, there are considerable drawbacks to overcome yet, including the interpretation of machine learning models and the development of robust models to account for disease and population heterogeneity. In fact, to date, with a given, specific dataset there is no gold standard for the selection of the optimal algorithm to be used. In fact, minimum changes in the dataset structure may result in significantly diverse outcomes as a function of the chosen algorithm. Further research should be conducted to better understand how algorithms interact with the characteristics of datasets.

## Figures and Tables

**Table 1:** Recent studies carried out to discover biomarkers combining metabolomics data and machine learning algorithms. Feature selection algorithms, number of purposed biomarkers, and type of disease are reported.

Feature Selection Algorithm	Number of Purposed Biomarkers	Study	Diseases
Random Forest (VIP score)-based features	18 metabolites as biomarker	[36]	Weight gain (Metabolomic disorder)
Fast correlation-based feature selection (FCBS)	5 biomarkers	[37]	Lung cancer
Recursive feature elimination and PLS regression	10 metabolites as biomarker	[38]	Renal cell carcinoma
MUVR algorithm	13 metabolites	[39]	Gout and asymptomatic hyperuricemia
PLSDA	26 Metabolites and lipids	[40]	Rheumatoid arthritis
SVM-RFE (Recursive feature elimination)	16 Metabolites	[41]	Epithelial Ovarian cancer

**Table 2:** Recent application of machine learning algorithms coupled to metabolomics for the diagnosis of diseases. The diseases, the best-performing algorithm and the samples analyzed are reported.

Diseases	Best-Performing Algorithms	Other Models	References	Sample Collection
Zika virus	RF	SVM (Sequential minimal optimization) and (Iterative single data algorithm), Decision Trees	[35]	Serum
Colorectal cancer	RF	PLS, LDA, SVM	[44]	Urine
Paracoccidioidomycosis	RF	N/A	[46]	Serum
Malignant Mesothelioma	RF	N/A	[47]	Plasma
Diabetic cognitive impairments	SVM	N/A	[53]	Urine
Benzylpenicillin and multidrug resistance of Staphylococcus aureus	SVM (Radial basis function), Logistic regression, Neural network	Random Forest, Linear SVM, ADA Boost, Quadratic discriminant analysis (QDA) and linear discriminant analysis (LDA), Naïve Bayes, Decision Tree	[54]	Milk
Intrauterine growth restriction	SVM (Radial basis function)	N/A	[55]	Cord blood serum
Parkinson Disease	Neural Network	N/A	[57]	Plasma from Blood
Small-cell lung cancer (SCLC) and non-small-cell lung cancer	Neural Network	N/A	[59]	Sputum
Lung cancer	Naïve Bayes	Random Forest, SVM, Neural Network, KNN, AdaBoost	[37]	Plasma
Renal Cell Carcinoma Status Prediction	KNN (k-nearest neighbor)	Random forest (RF), linear kernel support vector machine (SVM-Lin)	[38]	Urine
Gout from asymptomatic hyperuricemia	SVM	Logistic regression, Random Forest	[39]	Serum
Irritable Bowels Syndrome	Combination of Logistic regression and Random Forest	Random Forest, Logistic regression	[48]	Faecal samples
Autoimmune diseases	Artificial neural network and Logistic regression	NA	[61]	Plasma
Multiple sclerosis	Random Forest	GLM, PLS-DA, PCA-LDA	[49]	Plasma
Intracerebral hemorrhage from Acute Cerebral Infarction	Neural Network	N/A	[60]	Dried blood spot (DBS)

**Table 3:** Tools and libraries used by metabolomics studies for the application of machine learning algorithms.

Tools/Libraries	Purpose of Use in Studies	Programing Language	Programing Skills Requirement	Metabolomic Studies
Weka	Classification/feature selection	Java	No	[83,87]
KNIME	Data processing	Java	No	[89,90]
Orange data mining	Classification	Python, Cython, C++, C	No	[91]
Scikit-learn	Data processing/Classification	Python	Yes	[38,92]
TPOT	Classification/feature selection	Python	Yes	[93]
Caret	Classification/feature selection	R	Yes	[62,94]
Keras and Tensor flow	Data processing/Peak identification	Python, R	Yes	[95]
